# Stable Isotopic Tracing—A Way Forward for Nanotechnology

**DOI:** 10.1289/ehp.9277

**Published:** 2006-06-23

**Authors:** Brian Gulson, Herbert Wong

**Affiliations:** 1 Graduate School of the Environment, Macquarie University, Sydney, New South Wales, Australia; 2 Commonwealth Scientific and Industrial Organisation, Exploration and Mining, North Ryde, New South Wales, Australia

**Keywords:** isotope tracers, nanoparticles, quantum dots, sunscreen, titanium dioxide, zinc oxide

## Abstract

Numerous publications and reports have expressed health and safety concerns about the production and use of nanoparticles, especially in areas of exposure monitoring, personal use, and environmental fate and transport. We suggest that stable isotopic tracers, which have been used widely in the earth sciences and in metabolic and other health-related studies for several decades, could be used to address many of these issues. One such example we are pursuing is the use of stable isotopes to monitor dermal absorption of zinc and titanium oxides in sunscreen preparations and other personal care products. Other potential applications of this tracing approach are discussed.

Nanotechnology is currently one of the fastest growing technical fields that develops methods for the design, production, and applications for materials and devices ≤100 nm in size. There are numerous potential applications arising from nanotechnology which include their use in aerospace, agriculture, security, energy, information technology, medicine, transportation, consumer products, and environmental improvement (Nanoscale Science 2004). Nanotechnology is projected to be a US$ 1 trillion industry by 2015 (Nel et al. 2006).

Nanoparticles are particles that have one or more dimensions ≤100 nm [Scientific Committee on Emerging and Newly Identified Health Risks (SCENIHR) 2005]. Because of their small size, much concern has been expressed about the potential for adverse health effects arising from the ability of nanoparticles to penetrate cell walls and the blood–brain barrier. These concerns include possible detrimental health effects during manufacture and transport as well as their fate and transport in the environment [United Kingdom Royal Society 2004; U.S. Environmental Protection Agency (U.S. EPA) 2005]. At this time there are no occupational health and safety guidelines for production and use of these nano products. This concern over lack of guidelines has resulted in a coordinated collaborative effort between several U.S. agencies—the National Institutes of Health, the National Toxicology Program headquartered at the National Institute of Environmental Health Sciences, the U.S. EPA, the Centers for Disease Control and Prevention, the National Institute for Occupational Safety and Health, and the Occupational Safety and Health Administration—and comprehensive reports by The Royal Society and the Royal Academy of Engineering of the United Kingdom (2004), a European Commission report (SCENIHR 2005), and a White paper from the U.S. EPA (2005). These efforts address several additional critical issues that include the lack of standardization of materials available for confirmatory product evaluation, monitoring in the workplace and environment, and testing procedures.

Some of the major industries using or planning to use nanotechnology manufactured goods include electronics, personal care products, and metal and ceramic manufacturers. By design, many of the nanotechnology products in development or in use contain a metal (or metalloid in the case of arsenic) (Table 1). We suggest that many of the concerns outlined above can be addressed with the approach of isotopic tracing, whereby a stable isotope of the element of interest is incorporated into the product, allowing any transfer to be easily detected using inductively coupled mass spectrometry (ICP-MS), high resolution ICP-MS, multi-collector ICP-MS, or thermal ionization MS (TIMS). This approach differs from tracing using radiolabeled metals, such as ^64^Cu, which generally have a short half-life (Michalet et al. 2005)

## Stable Isotope Tracing

For several decades, stable isotope tracing has been used widely in the earth sciences, such as understanding the origin of rocks and in environmental applications. This technique also has been applied in nutritional and metabolic balance studies and health investigations (Gulson 1996). In this commentary, we use the term “stable” for metal isotopes rather than its common application to the light-stable isotopes of hydrogen, carbon, nitrogen and sulfur.

There are two main approaches in the use of isotope tracing, one based on naturally occurring differences between stable isotopes and the other that uses the addition of a tracer of the separated isotope.

The first approach uses variations in isotopic abundance of the stable end products arising from radioactive decay. For example, the stable end products of lead-206 (^206^Pb), ^207^Pb, and ^208^Pb, are derived by long-term radioactive decay of parents uranium-238 (^238^U), ^235^U, and thorium-232, respectively. The fourth lead isotope, ^204^Pb, has no known radioactive parent. Hence, over geologic time, lead forms into mineral deposits that have major differences in isotopic composition, that can accumulate in the bones of the inhabitants. Such naturally occurring isotopic differences have been used in recent investigations of the mobilization of lead from the maternal skeleton during pregnancy and lactation (Gulson et al. 1997, 1998, 2004; Manton et al. 2003). In the Australian study (Gulson et al. 1997, 1998, 2004), investigators examined *a*) differences in the lead isotopic signature of long-term Australian residents and the environment exposed to geologically old mine lead formed > 1.7 billion years ago, and *b*) the signature in migrants to Australia who were generally exposed to geologically young lead formed approximately 300–400 million years ago. By monitoring blood and urine lead isotopic values during pregnancy and lactation and comparing these isotopic values with those in the environment (diet, air, water, soil, dust), it was possible to estimate the extra amount of lead coming from the maternal skeleton. Similarly, variations in the four isotopes of strontium (^84^Sr, ^86^Sr, ^87^Sr, ^88^Sr) have been used to investigate migration paths and dietary habits of humans and animals (Beard and Johnson 2000).

The second approach applies to elements that do not have a radioactive parent. In this case, there are negligible or very small variations in natural abundance between the different isotopes. To trace such elements, a stable non-radioactive isotope or tracer whose abundance is different from that occurring naturally is incorporated into the product. Many tracers are available from commercial suppliers and their price usually depends on the natural abundance, with the lower the natural abundance the higher the cost. This technique can be applied easily for some of the elements and compounds currently being designed or used as nanoparticles (Table 1). Where possible, if not cost-prohibitive, the tracer can be added to enhance the isotopic abundance to show a difference from that occurring naturally, thus making it easier to detect in a study. The ability to detect differences depends on a number of factors, including the purity of the separated tracer, the total amount of the element under investigation, and the sensitivity of the instruments for measuring the isotopic abundances. For example, TIMS is considered to be the “gold standard” for isotopic measurements although multi-collector ICP-MS or high-resolution ICP-MS may also be suitable, depending on conditions. This approach has been used widely in nutritional and metabolic balance studies [see references up to 1995 in Gulson (1996)] and pharmacokinetic investigations of, for example, lead in humans (Rabinowitz et al. 1976) and cynomolgous monkeys (Franklin et al. 1997).

## Possible Uses for Isotopic Tracing in Nanotechnology

The second approach, which uses isotopic tracers and is described in the previous section, offers the greatest potential for use in nanotechnology. For example, zinc oxide (ZnO) and titanium dioxide (TiO)nanoparticles are being used in sunscreens and other personal care products. Zinc has five isotopes whose naturally occurring abundances are listed in Table 1. ^68^Zn with a natural abundance of 18.6% is the isotope of choice for studies of zinc oxide because of cost considerations. We are conducting investigations using ^68^Zn and ^46^Ti as tracers in sunscreen products containing zinc oxide and titanium dioxide to determine *in vivo* their dermal absorption and excretion in humans.

Use of zinc isotopes is not just limited to sunscreen nanoparticles, as zinc is found in several other nanoparticles such as quantum dots. Quantum dots are crystals of semiconductor compounds [e.g., cadmium selenide (CdSe), lead sulfide] with a diameter between 2- and 10-nm wide or 10 and 50 atoms, respectively, and have a range of useful electrical and optical properties that diverge in character from those of bulk material (Evident Technologies Inc. 2006). For example, quantum dots are being researched as potential artificial fluorophores for detection of tumors, using fluorescence spectroscopy. In one product being developed, the metals giving rise to the fluorescence of CdSe or cadmium telluride (CdTe), have a coating of zinc sulfide to minimize exposure to the highly toxic Cd and potentially toxic Se or Te. There is concern that the coating may degrade before the quantum dots are excreted from the body, but apart the data from mice and cellular studies, there are no human data (Derfus et al. 2004; Lovric et al. 2005). The integrity of the coating could be monitored by using either a single isotopic tracer such as ^68^Zn incorporated into the coating or an “overkill” that could employ a multiple isotopic approach, as Cd has eight naturally occurring isotopes, Se has six, Te has eight, and Zn has five (Table 1).

Stable isotope tracing for monitoring exposures to nanoproducts has many potential uses, including incorporation of a tracer in the production process to monitor worker exposure. The exposure can be monitored from isotopic measurements of wipes from areas such as protective clothing, hands, and face, or collection of biomarkers such as blood and urine. Furthermore, simple surface wipes and/or dust accumulation methods using petri dishes could be used for air monitoring exposures, followed by isotopic analysis.

In addition to the occupational aspects, concerns over fate and transport of nanoparticles in the environment (U.S. EPA 2005) and pharmacokinetic modeling and cytotoxicity studies such as those undertaken by the National Toxicology Program (Walker 2006) also could be addressed using the isotopic approach.

## Conclusions

We suggest that the stable isotope tracing approach has enormous capability to address many of the current concerns (noted in the introduction) being raised by scientific bodies. The stable isotope tracing approach has many advantages for use for monitoring purposes in the nanotechnology field, including *a*) many of the metals/metalloids currently used in nanotechnology have more than one isotope, and this approach can be readily implemented; *b*) the stable, usually high-purity, isotopes used in such studies are nonradioactive; *c*) the stable isotopes do not have the disadvantage of radioactive tracers which commonly have very short half-lives and may give low radiation doses, and thus allow long-term monitoring; and *d*) the methods for isotopic measurements of ICP-MS and TIMS are applicable to most metals and are routinely used in environmental and health investigations. While the initial investment to obtain the isotopic tracers may be high, the dividends of providing reliable scientific evaluations for monitoring these nanoparticles in environmental and biological systems will more than offset the initial cost.

## Figures and Tables

**Table 1:** Some products used in nanotechnology and their natural relative isotopic abundances.

Product	Symbol	Isotope and natural relative abundances (%)
Cadmium sulfide	CdS	Cd: 106 (1.2), 108 (0.9), 110 (12.4), 111 (12.8), 112 (24.0), 113 (12.3), 114 (28.8), 116 (7.6)
		S: 32 (95.0), 33 (0.75), 34 (4.2), 36 (0.02)
Cadmium selenide	CdSe	Se: 74 (0.9), 76 (9.0), 77 (7.5), 78 (23.5), 80 (50), 82 (9)
Cadmium telluride	CdTe	Te: 120 (0.09), 122 (2.4), 123 (0.87), 124 (4.6), 125 (7.0), 126 (18.7), 128 (31.8), 130 (34.5)
Calcium	Ca	Ca: 40 (96.9), 42 (0.65), 43 (0.14), 44 (2.08), 46 (0.003), 48 (0.19)
Chromium	Cr	Cr: 50 (4.35), 52 (83.8), 53 (9.5), 54 (2.36)
Iron	Fe	Fe: 54 (5.8), 56 (91.7), 57 (2.14), 58 (0.31)
Gallium phosphide/arsenide	GaP/As	Ga: 69 (60), 71 (40)
Gallium antimonide/selenide/telluride	GaSb/Se/Te	Sb:121 (57.3), 123 (42.7)
Indium phosphide/arsenide/antimonide	InP/As/Sb	In: 113 (4.3), 115 (95.7)
Lead sulfide/selenide/telluride	PbS/Se/Te	Pb: 204 (1.4), 206 (24.1), 207 (22.1), 208 (52.4)
Magnesium	Mg	Mg: 24 (79), 25 (10), 26 (11)
Molydenum	Mo	Mo: 92 (14.8), 94 (9.1), 95 (15.9), 96 (16.7), 97 (9.5), 98 (24.4)
Silicon germanium/carbide	SiGe/C	Si: 28 (92.2), 29 (4.7), 30 (3.1);Ge: 70 (20.7), 72 (27.5), 73 (7.7), 74 (36.4), 76 (7.7)
Tantalum	Ta	Ta: 180 (0.01), 181 (99.9)
Silver	Ag	Ag: 107 (51.4), 109 (48.7)
Titanium dioxide	TiO_2_	Ti: 46 (8.0), 47 (7.5), 48 (73.7), 49 (5.5), 50 (5.3)
Tungsten	W	W: 180 (0.13), 182 (26.3), 183 (14.3), 184 (30.7), 186 (28.6)
Vanadium	V	V: 50 (0.25), 51 (99.7)
Zinc oxide/sulfide/selenide/telluride	ZnO/S/Se/Te	Zn: 64 (48.9), 66 (27.8), 67 (4.1), 68 (18.6), 70 (0.62)
Zirconium	Zr	Zr: 90 (51.4), 91 (11.2), 92 (17.1), 94 (17.5), 96 (2.8)
